# Syntheses, Structures and Biological Activites of Organogermaniun and Organotin Derivatives of Hydroxamic Acids

**DOI:** 10.1155/MBD.1998.237

**Published:** 1998

**Authors:** Valery S. Petrosyan, Natalia S. Yashina, Sergei V. Ponomarev

**Affiliations:** Department of Chemistry M. V. Lomonosov University Moscow 119899 Russia


### SYNTHESES, STRUCTURES AND BIOLOGICAL ACTIVITIES OF ORGANOGERMANIUM AND ORGANOTIN DERIVATIVES OF HYDROXAMIC ACIDS

Valery S. Petrosyan*, Natalia S. Yashina, and Sergei V. Ponomarev

Department of Chemistry, M. V. Lomonosov University, Moscow 119899, Russia

Hydroxamic acids as the precursors of their organogermanium and organotin derivatives have been themselves intensively studied from the viewpoint of their potential biological activities [1-3]. The wide spectrum of biological activities, from fungicide, bactericide and antiflammatory to psychotropic and antitumor, has been demonstrated for various organic derivatives of hydroxamic acids.

Mironov and coworkers [4] at first obtained the C-germylated hydroxamic acids with about 70% yield by the reaction of the methyl ester of 3-trimethylgermylpropionic acid with hydroxylamine chlorohydrate:

$$Me_3GeCH_2CH_2COOCH_3 + NH_2OH \cdot HCl \xrightarrow{0^oC} Me_3GeCH_2CH_2CONHOH \cdot HCl \xrightarrow{KOH}$$

$$\longrightarrow Me_3GeCH_2CH_2CONHOH$$

They have expanded later [5,6] this method to obtain some other β-germylated hydroxamic acids:

$$R_3GeCH_2CH(R')COOMe \xrightarrow{NH_2OH, KOH} R_3GeCH_2CH(R')CONHOK \xrightarrow{HCl, H_2O}$$

$$\longrightarrow R_3GeCH_2CH(R')CONHOH$$

$R_3Ge = Me_3Ge, Et_3Ge, I\text{-}AdMe_2Ge; R' = H, Me$

The attempt to obtain α-germylacetohydroxamic acid was not successful, possibly due to the β-elimination processes both in starting material and in final product:

$$Me_3GeCH_2COOMe + NH_2OH \cdot HCl \xrightarrow{KOH} MeCONHOH + Me_3GeOMe$$

It has been shown also [7] that the acylation of tris(trimethylsilyl)hydroxylamine with 3-trimethylgermylpropionyl and 4-triethylgermylbutyryl chlorides yields in the corresponding hydroxamic acids:

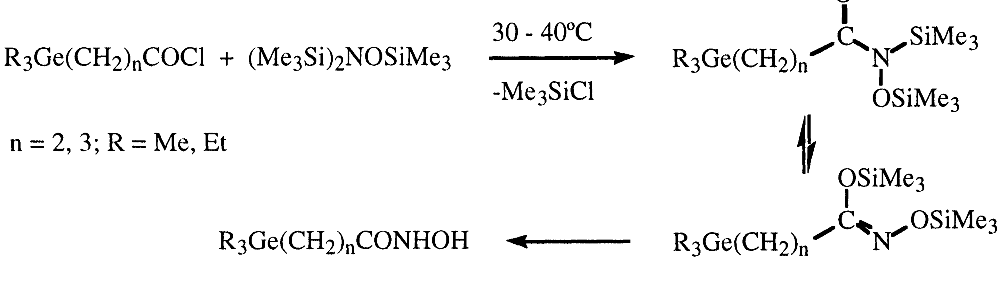

Diacylation products were not formed.

Table 1. The bond distances and angles in structure of β-triethylgermylpropiohydroxamic acid (I)

| Atoms • | d,,Å A | d,,Å B | Atoms | ω , ° A | ω , ° B |
|---|---|---|---|---|---|
| Ge-C(1) | 1.91(1) | 1.93(4) | C(1)GeC(4) | 108(1) | 108(1) |
| Ge-C(4) | 1.96(6) | 1.93(6) | C(1)GeC(6) | 109(1) | 111(1) |
| Ge-C(6) | 1.91(4) | 1.92(4) | C(1)GeC(8) | 107(2) | - |
| Ge-C(8) | 2.08(5) | 2.13(5) | C(4)GeC(6) | 109(1) | 111(1) |
| O(1)-C(3) | 1.24(4) | 1.25(4) | C(4)GeC(8) | 114(2) | - |
| O(2)-N | 1.38(4) | 1.36(4) | C(6)GeC(8) | 110(2) | - |
| N-C(3) | 1.34(6) | 1.32(5) | O(2)NC(3) | 119(2) | 120(2) |
| C(1)-C(2) | 1.48(7) | 1.49(7) | GeC(1)C(2) | 118(1) | 116(1) |
| C(2)-C(3) | 1.49(7) | 1.50(7) | C(1)C(2)C(3) | 115(2) | 114(1) |
| C(4)-C(5) | 1.49(9) | 1.54(9) | O(1)C(3)N | 124(2) | 124(2) |
| C(6)-C(7) | 1.52(9) | 1.46(9) | O(1)C(3)C(2) | 123(1) | 121(2) |
| C(8)-C(9) | 1.16(9) | 1.36(9) | NC(3)C(2) | 113(2) | 116(1) |
| | | | GeC(4)C(5) | 116(2) | 114(2) |
| | | | GeC(6)C(7) | 119(2) | 119(1) |
| | | | GeC(8)C(9) | 124(3) | - |

Mehrotra and coworkers [8] have studied in details the O-germylation of salicylhydroxamic acid. They have shown that the nature of the product depends substantially on the ratio of reagents:

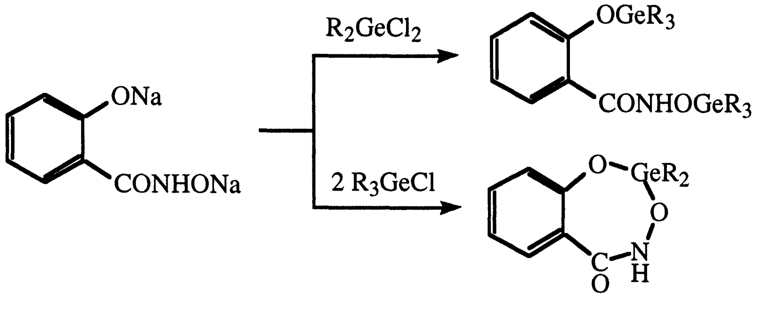

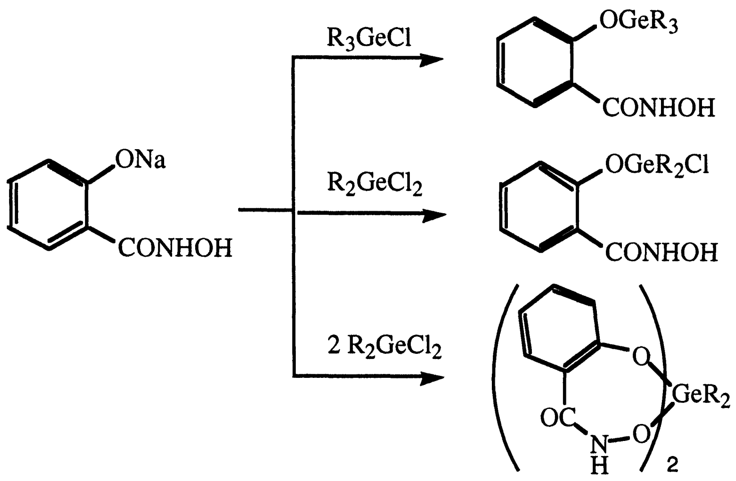

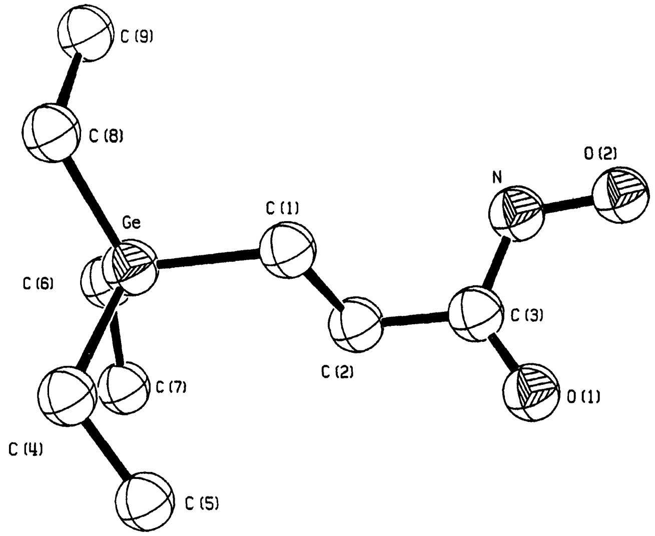

(I)

Figure 1: Structure and atomic numbering scheme for $Et_3GeCH_2CH_2CONHOH$ (I)

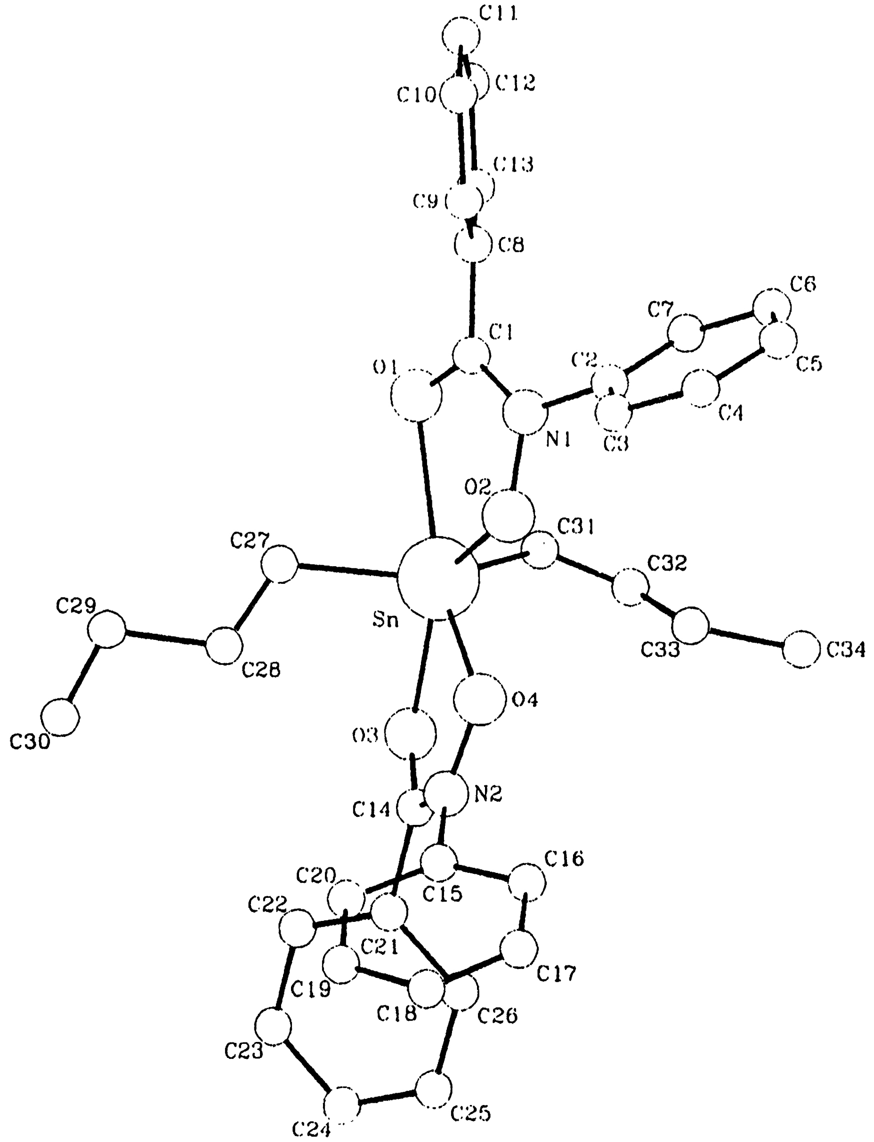

Figure 2: Structure and atomic numbering scheme for $(n\text{-}Bu_2Sn(ON(Ph)C(O)Ph)_2$ (II)

As to the structures of organogermanium derivatives of hydroxamic acids, not very much data were published and we could find at least one structure of β-triethylgermylpropiohydroxamic acid (I) in the Ph. D. Thesis of Feoktistov.
The bond distances and angles in two crystallographically independent molecules A and B are given in Table 1. The biological activities of some organogermanium derivatives of hydroxamic acids have been studied by Lukevics and coworkers [10]. It has been shown that $(CH_3)_3GeCH_2CH(R')CONHOH$ (R' = H, Me) have rather high level of antihypoxy activity. 3-(Adamantyldimethylgermyl)propiohydroxamic acid is exhibiting antihypoxy neurotropic activity with relatively low toxicity.

Table 2. Selected bond distances and angles in structure II.

| Atoms | d, Å | Atoms | ω(°) | Atoms | ω(°) |
|---|---|---|---|---|---|
| Sn-O(1) | 2.424(6) | O(1)SnO(2) | 70.4(2) | SnO(1)C(1) | 110.6(4) |
| Sn-O(2) | 2.088(5) | O(1)SnO(3) | 146.0(2) | SnO(2)N(1) | 118.1(4) |
| Sn-)(3) | 2.416(6) | O(1)SnO(4) | 143.2(2) | SnO(3)C(14) | 111.0(5) |
| SnO(4) | 2.084(5) | O(1)SnC(27) | 78.3(3) | SnO(4)N(2) | 116.9(4) |
| Sn-C(27) | 2.149(9) | O(1)SnC(31) | 83.9(3) | O(2)N(1)C(1) | 117.7(6) |
| Sn-C(31) | 2.115(12) | O(2)SnO(3) | 143.4(2) | O(2)N(1)C(2) | 113.5(5) |
| O(1)-C(1) | 1.253(10) | O(2)SnO(4) | 73.8(2) | C(1)N(1)C(2) | 127.8(7) |
| O(2)-N(1) | 1.383(8) | O(2)SnC(27) | 108.9(4) | O(4)N(2)C(14) | 119.0(6) |
| O(3)-C(14) | 1.251(10) | O(2)SnC(31) | 104.3(3) | O(4)N(2)C(15) | 112.1(5) |
| O(4)-N(2) | 1.390(8) | O(3)Sn)O(4) | 70.6(2) | C(14)N(2)C(15) | 128.4(7) |
| N(1)-C(1) | 1.334(9) | O(3)SnC(27) | 88.6(4) | O(1)C(1)N(1) | 120.2(7) |
| N(1)-C(2) | 1.417(9) | O(3)SnC(31) | 83.0(4) | O(1)C(1)C(8) | 120.0(6) |
| N(2)-C(14) | 1.324(10) | O(4)SnC(27) | 105.6(3) | N(1)C(1)C(8) | 119.7(7) |
| N(2)-C(15) | 1.453(10) | O(4)SnC(31) | 113.7(3) | O(3)C(14)N(2) | 119.2(7) |
| C(1)-C(8) | 1.478(11) | C(27)SnC(31) | 133.9(4) | O(3)C(14)C(21) | 121.8(7) |
| C(14)-C(21) | 1.486(11) | | | N(2)C(14)C(21) | 119.0(7) |

As to the biological activities of organotin derivatives of hydroxamic acids, we have to mention first of all that Lukevics and coworkers [10] in their study have shown that the highest neurotropic activity has been exhibited by β-trimethylstannylpropiohydroxamic acid, $Me_3SnCH_2CH_2CONHOH$, which together with $Me_3SnCH_2CH(Me)CONHOH$ have been showing the highest level of antihypoxy activity as well.
In recent years the syntheses, physico-chemical properties and structures of organotin hydroxamates have been the subject of a number of studies [11-20]. As a rule, the hydroxamate ligand behaves as bidentate because of the carbonyl coordination to tin, thus causing a substantial reduction in the infrared carbonyl stretching frequencies in comparison to the free hydroxylamines [11-15]. On the other hand, the definitive conclusion about the organotin hydroxamate structure could be made basing on the Mössbauer parameters by means of their correlation with appropriate crystal structure parameters [11, 12]. However, only a few X-ray structure determinations of organotin N-acylhydroxylamine derivatives are available in the literature [16-19].
In further discussion we will concentrate on the results of structural studies in the solid state and in solution of bis (N-phenyl-N-benzoylhydroxylamino)di-n-butyltin, $n\text{-}Bu_2Sn(ON(Ph)C(O)Ph)_2$, (II), diorganotin derivatives of N-methyl-N-p-bromobenzoylhydroxylamine, $R_2Sn\{ON(Me)C(O)C_6H_4Br\text{-}p\}_2$, R = Me (III), Et(IV), Bu(V), Ph(VI) and N-p-bromophenyl-N-p-bromobenzoylhydroxylamine, $R_2Sn\{ON(C_6H_4Br\text{-}p)C(O)C_6H_4Br\text{-}p\}_2$, R = Me (VII), Et (VIII), Bu (IX) and Ph (X), of which (II) was prepared for the first time by Harrison and Richards [11]. The hydroxamates were characterized by IR, Mössbauer and NMR ($^1H$, $^{13}C$, $^{119}Sn$) spectroscopy as well as by an X-ray diffraction analysis for (II), (IV) and (V).
The structures and atomic numbering schemes for II, IV and V are depicted in figs. 1, 2 and 3, selected bond distances and angles are reported in Tables 2 – 4. The geometry around the tin atom is similar to that found for some other diorganotin bis-hydroxamates [16, 17]. In the structures under consideration, the two N-acylhydroxylamine residues are almost equivalent and behave as bidentate ligands, forming one short covalent and one longer coordination oxygen-to-tin bond . The two heterocyclic rings are almost planar, the deviation of tin from these planes being 0.45 Å and 0.49 Å for II, 0.09 Å and 0.04 Å for V and 0.01 Å for

IV. The phenyl rings are rotated by 45-60° relative to the planes of the N-acylhydroxylamine residues. The CSnC fragment is not linear within these structures, the C-Sn-C angles being 133.9° for II , 141,1° for IV. and 145.1° for V. This geometry at tin is usually described as distorted trans-octahedral [11, 16, 17].

Table 3. Selected bond distances and angles in structure IV.

| Atoms | d, Å | Atoms | ω (°) | Atoms | ω, (°) |
|---|---|---|---|---|---|
| Sn-O(1) | 2.375(5) | O(1)Sn(1)O(2) | 140.7(2) | Sn(1)O(1)C(1) | 110.6(4) |
| Sn-O(2) | 2.413(5) | O(1)Sn(1)O(10) | 72.2(2) | Sn(1)O(2)C(2) | 111.0(4) |
| Sn-O(10) | 2.102(5) | O(1)Sn(1)O(20) | 148.4(2) | Sn(1)O(10)N(1) | 116.1(4) |
| Sn-O(20) | 2.106(5) | O(1)Sn(1)C(3) | 83.8(3) | Sn(1)O(20)N(2) | 117.0(4) |
| Sn-C(3) | 2.109(10) | O(1)Sn(1)C(5) | 83.6(3) | O(1)C(1)N(1) | 121.7(6) |
| Sn-C(5) | 2.117(10) | O(2)Sn(1)O(10) | 147.1(2) | O(1)C(1)O(11) | 119.2(6) |
| O(1)-C(1) | 1.242(8) | O(2)Sn(1)O(20) | 70.9(2) | O(2)C(2)N(2) | 119.8(6) |
| O(2)-C(2) | 1.259(8) | O(2)Sn(1)C(3) | 82.9(3) | O(2)C(2)C(21) | 119.7(6) |
| O(10)-N(1) | 1.386(7) | O(2)Sn(1)C(5) | 84.0(3) | O(10)N(1)C(1) | 119.2(6) |
| O(20)-N(2) | 1.377(7) | O(10)Sn(1)O(20) | 76.3(2) | O(10)N(1)C(10) | 112.0(6) |
| N(1)-C(1) | 1.314(8) | O(10)Sn(1)C(3) | 104.1(3) | O(20)N(2)C(2) | 120.0(6) |
| N(1)-C(10) | 1.453(10) | O(10)Sn(1)C(5) | 106.8(3) | O(20)N(2)C(20) | 112.0(6) |
| N(2)-C(2) | 1.313(8) | O(20)Sn(1)C(3) | 106.1(3) | N(1)C(1)C(11) | 119.2(6) |
| N(2)-C(20) | 1.452(9) | O(20)Sn(1)C(5) | 103.9(4) | N(2)C(2)C(21) | 120.4(6) |
| C(1)-C(11) | 1.511(9) | C(3)Sn(1)C(5) | 141.1(4) | C(1)N(1)C(10) | 128.2(6) |
| C(2)-C(21) | 1.483(9) | | | C(2)N(2)C(20) | 127.6(6) |

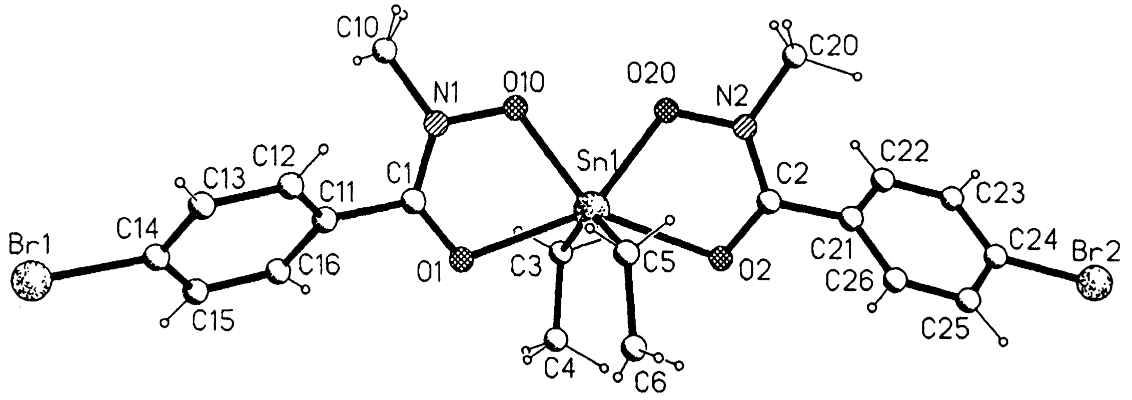

Figure 3: Structure and atomic numbering scheme for $(Et_2Sn(ON(Me)C(O)C_6H_4Br\text{-}p)_2$ (IV)

As in other organotin hydroxamates [16-18], bond distances in the N-acylhydroxylamine residues are consistent with a significant contribution of the zwitterionic canonical form to the electronic distribution, the C=O distance being longer and the endocyclic C-N distance shorter than normal C=O double bond and C-N single bond distances, respectively [14].

In the IR spectra of all solid compounds discussed, the carbonyl stretching vibrations occur at lower frequencies (Table 5) in comparison to the spectra of the free hydroxylamines, [HON(Ph)C(O)Ph, 1624 $cm^{-1}$, $HON(Me)C(O)C_6H_4$-p-Br, 1610 $cm^{-1}$ and $HON(C_6H_4$-p-Br)$C(O)C_6H_4$-p-Br, 1602 $cm^{-1}$] confirming again the bidentate behavior of the hydroxylamine residues .in the hydroxamates [11-16].

The Mössbauer parameters (isomer shifts IS and quadrupole splittings QS) of the hydroxamates under the discussion are given in Table 5. According to the X-ray crystal data the distortion of their trans-octahedral structures is so great that a traditional comparison of experimental values $QS_{exp}$ with calculated ones $QS_{cal}$ in terms of the point charge model formalism assuming idealized octahedral environment around tin seems to be pointless. For comparison we have made such calculation using literature values for the partial quadrupole splittings of the alkyl and ligand groups and real angle values determined from X-ray analysis of V (Table 4). The resulting $QS_{calc}$ = 3.09 $mm.s^{-1}$ is in good agreement with $QS_{exp}$ of compounds II, IV, V, VII , VIII and $Me_2Sn(ON(Me)C(O)Me)_2$ (QS = 3.31 $mm.s^{-1}$ [11]), for which a distorted trans-octahedral structure was established [16]. Thus it is logically basing on Mössbauer spectroscopy data to ascribe the distorted octahedral structure with alkyl groups in trans-position to each other also to hydroxamates VII and VIII.

Table 4. Selected bond distances and angles in structure V.

| Atoms | d, Å | Atoms | ω(°) | Atoms | ω(°) |
|---|---|---|---|---|---|
| Sn-O(1) | 2.101(4) | O(1)SnO(2) | 73.9(1) | SnO(2)N(2) | 118.2(3) |
| Sn-O(2) | 2.114(3) | O(1)SnO(3) | 72.2(1) | SnO(3)C(2) | 111.5(3) |
| Sn-O(3) | 2.364(4) | O(1)SnO(4) | 144.2(1) | SnO(4)C(10) | 112.3(4) |
| Sn-O(4) | 2.393(4) | O(1)SnC(17) | 103.3(2) | O(1)N(1)C(1) | 111.5(4) |
| Sn-C(17) | 2.118(6) | O(1)SnC(21) | 107.1(2) | O(1)N(1)C(2) | 119.8(4) |
| Sn-C(21) | 2.117(6) | O(2)SnO(3) | 145.9(1) | C(1)N(1)C(2) | 128.5(5) |
| O(1)-N(1) | 1.382(5) | O(2)SnO(4) | 70.7(1) | O(2)N(2)C(9) | 111.7(4) |
| O(2)-N(2) | 1.376(6) | O(2)SnC(17) | 103.9(2) | O(2)N(2)C(10) | 118.6(4) |
| O(3)-C(2) | 1.260(7) | O(2)SnC(21) | 100.7(2) | C(9)N(2)C(10) | 128.9(5) |
| O(4)-C(10) | 1.248(6) | O(3)SnO(4) | 143.4(1) | O(3)C(2)N(1) | 120.1(5) |
| N(1)-C(1) | 1.448(8) | O(3)SnC(17) | 86.8(2) | O(3)C(2)C(3) | 119.2(5) |
| N(1)-C(2) | 1.317(7) | O(3)SnC(21) | 86.5(2) | N(1)C(2)C(3) | 120.7(5) |
| N(2)-C(9) | 1.434(7) | O(4)SnC(17) | 81.1(2) | O(4)C(10)N(2) | 120.2(5) |
| N(2-C(10) | 1.326(7) | O(4)SnC(21) | 84.1(2) | O(4)C(10)C11) | 119.6(5) |
| C(2)-C(3) | 1.481(7) | C(17SnC(21) | 145.1(3) | N(2)C(10)C(11) | 120.1(5) |
| C(10)-C(11) | 1.489(8) | SnO(1)N(1) | 116.4(3) | | |

**Table 5.** Mössbauer parameters and infrared carbonyl stretching frequencies for diorganotin bis-hydroxamates.

| Compound | $IS^a$ ($mm.s^{-1}$) | $QS^b$($mm.s^{-1}$) | ν(CO)($cm^{-1}$) |
|---|---|---|---|
| n-$Bu_2Sn(ON(Ph)C(O)Ph)_2$, (II)* | 1.28 | 3.10 | 1553,1562sh |
| $Me_2Sn\{ON(Me)C(O)C_6H_4Br\text{-}p\}_2$, (III) | 0.87 | 2.13 | 1579 |
| $Et_2Sn\{ON(Me)C(O)C_6H_4Br\text{-}p\}_2$, (IV) | 1.28 | 3.39 | 1590 |
| $Bu_2Sn\{ON(Me)C(O)C_6H_4Br\text{-}p\}_2$, (V) | 1.28 | 3.27 | 1580 |
| $Ph_2Sn\{ON(Me)C(O)C_6H_4Br\text{-}p\}_2$, (VI) | 0.73 | 1.66 | 1587 |
| $Me_2Sn\{ON(C_6H_4Br\text{-}p)C(O)C_6H_4Br\text{-}p\}_2$, (VII) | 1.23 | 3.34 | 1542 |
| $Et_2Sn\{ON(C_6H_4Br\text{-}p)C(O)C_6H_4Br\text{-}p\}_2$, (VIII) | 1.27 | 3.30 | 1543 |
| $Bu_2Sn\{ON(C_6H_4Br\text{-}p)C(O)C_6H_4Br\text{-}p\}_2$, (IX) | 1.26 | 2.90 | 1533 |
| $Ph_2Sn\{ON(C_6H_4Br\text{-}p)C(O)C_6H_4Br\text{-}p\}_2$, (X) | 0.78 | 1.87 | 1528 |

*literature data : IS 1.34 $mm.s^{-1}$, QS 3.30 $mm.s^{-1}$, ν(CO) 1552, 1564 $cm^{-1}$[3]

However, the dimethyltin derivative III has substantially lower $QS_{exp}$ = 2.13 mm/s. Principally such low QS value can correspond to a tetrahedral structure for this compound, but it is difficult to expect a tetrahedral structure in this case, when the first coordination sphere of the tin is not sterically hindered by bulky R

groups, both donor centers of the ligand having comparable donating abilities. On the other hand the IR data of III are similar to that found for the di-n-butyltin derivative V having, in agreement with our X-ray analysis, a trans-octahedral chelate structure. Thus we suppose that the tetrahedral structure for III is unlikely and suggest for it a cis-octahedral structure, similar to that of $Me_2Sn(ONHC(O)Me)_2$ [17] with QS = 2.01 mm/s [11 ].

The diphenyltin derivatives VI and X have substantially lower QS values, viz. 1.66 and 1.87 $mm{\cdot}s^{-1}$). Principally, such low QS values can be correlated with a tetrahedral structures for these compounds, but the infrared data make this rather unlikely, since they are similar to those obtained for the trans-octahedral chelate diethyl- and dibutyltin analogs [20 a]. On this basis we suggest a cis-octahedral structure for both VI and X as suggested for some other diphenyltin bis-hydroxamates based on their Mössbauer parameters (QS = 1.61 - 1.95 $mm{\cdot}s^{-1}$[11, 12]).

The $^{1}H$, $^{13}C$ and $^{119}Sn$ NMR parameters of the compounds under consideration are reported in Table 6. The $^{1}H$ NMR spectra exhibit the expected proton signals in the correct integrated ratios. If we use the existing correlation between spin-spin coupling constants $^{1}J(^{13}C-^{119}Sn)$, $^{2}J(^{1}H-C-^{119}Sn)$ and C-Sn-C angle for methyltin(IV) compounds [21], it can be suggested that, after a dissolution of the compounds II, V, VII and IX in $CDCl_3$, the distorted trans-octahedral structure is preserved, as it has been proposed also for $Me_2Sn(ON(Me)C(O)Me)_2$ and $Me_2Sn(ON(Ph)C(O)Ph)_2$ [11].

It is interesting to mention that the $^{1}J(^{13}C-^{119}Sn)$ and $^{2}J(^{1}H-^{119}Sn)$ values of the compound III fall in the same range. We suggest that a structural change occurs upon dissolution of III in $CDCl_3$, and that the cis-octahedron transforms into a distorted trans-octahedron similarly to what was observed for $Me_2Sn(ONHC(O)Me)_2$ [11]. The $\delta^{119}Sn$ values (Table 6) of compounds II–X are typical for six-coordinate diorganotin compounds [22]

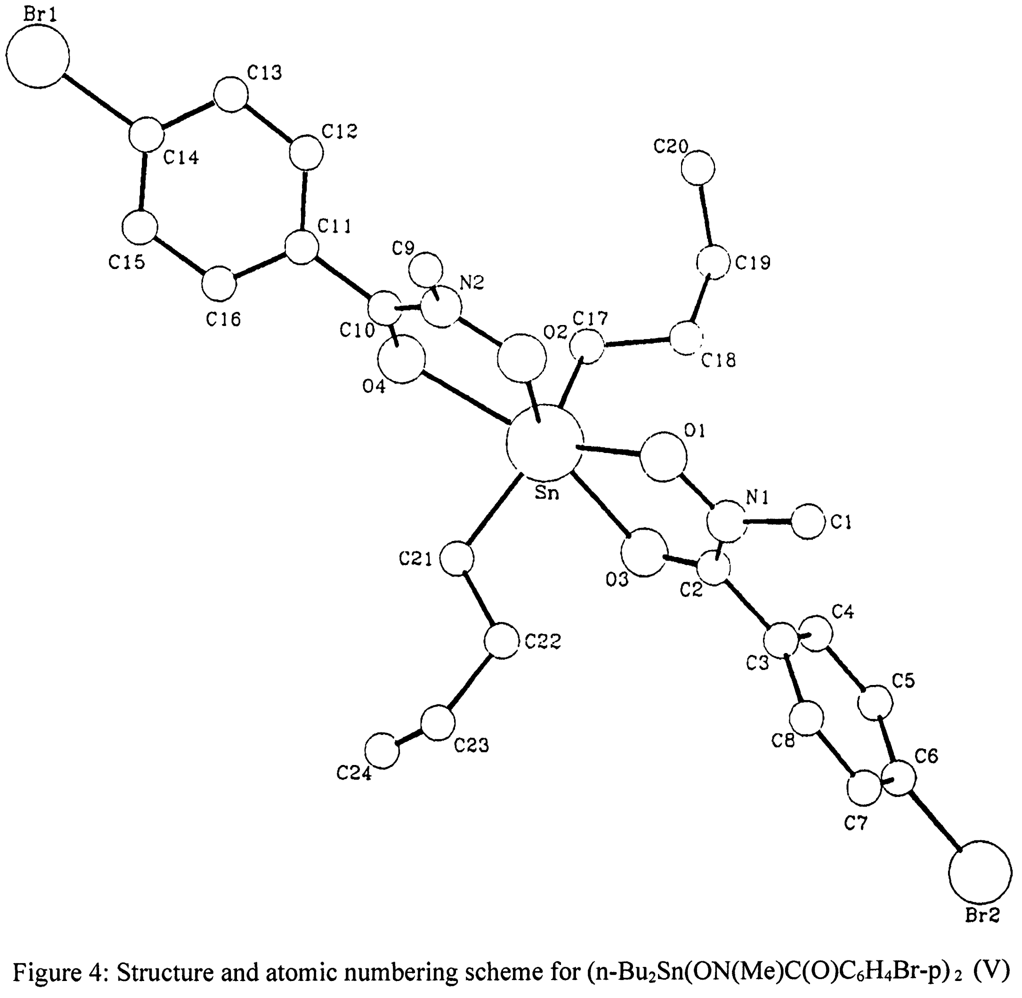

Figure 4: Structure and atomic numbering scheme for $(n-Bu_2Sn(ON(Me)C(O)C_6H_4Br-p)_2$ (V)

Table 6. Some NMR-spectroscopic data for diorganotin bis-hydroxamates (solvent $CDCl_3$).

| Structure | $CSnC^o_{,calc,}$ | $CSnC^o_{,expr}$ | $^2J(^1H-^{119}Sn)$ | $^1J(^{13}C-^{119}Sn)$ | $\delta^{119}Sn$ |
|---|---|---|---|---|---|
| II | 139 | 133.9 | nv | 709 | -226 |
| III | 143 | | 83.6 | 754 | -198 |
| IV | 144 | 141.1 | nv | 739 | -240 |
| V | 142 | 145.1 | nv | 739 | -236 |
| VI | | | nv | 925 | -361 |
| VII | 139 | | 81.2 | 713 | -182 |
| VIII | 141 | | nv | 730 | -227 |
| IX | 139 | | nv | 706 | -222 |
| X | | | nv | 919 | -354 |

**References.**

1. I.W. Munson , Chemistry and Biology of Hydroxamic Acids. Dayton, Ohio, USA, 1981. N.Y.: Karger Publ. Inc., 1982, p.1.
2. Z. Eckstein, T.Urbanski, *Wied. Chem.* **97** (1983) 347.
3. A.E.Feoktistov, V.F.Mironov, *Metalloorgan. Chimia* **3** (1990) 1207.
4. V.F.Mironov, A.E.Feoktistov, V.P.Kozynkov, *Zh.Obsch. Khim.* **55** (1985) 1385
5. O.N.Chernyshova, T.K.Gar, A.V.Kisin, V.F.Mironov, *Zh.Obsch. Khim* **55** (1985) 2333
6. A.E.Feoktistov V.F.Mironov, *Zh.Obsch. Khim* **58** (1988) 553
7. V.P.Kozynkov, A.E.Feoktistov, V.F.Mironov, *Zh.Obsch. Khim* **58** (1988) 1056
8. P.R.Shaudilya, G. Srivastsva, R.C.Mehrotra, *Indian J.Chem.* **25A** (1986) 155
9. A.E.Feoktistov, Ph.D.Thesis, Federal Research Institute of Organoelement Compounds Chemistry and Technology (GNIIKhTEOS), Moscow, 1987.
10. E. Lukevics, S.Germane, M. Trushule, A.E.Feoktistov, *Izv. AN Latv.SSR*, 1988, N5, p.79.
11. P.G.Harrison, J.A.Richards, *J. Organomet. Chem.* **185** (1989) 9
12. M.K.Das, M.Nath, J.J.Zuckermann, *Inorg.Chim.Acta* **71** (1983) 49.
13. B.Pradhan, A.K.Ghosh, *J.Organomet. Chem.* **131** (1977) 23.
14. M.K.Das, M.Nath, *Indian J.Chem.* **A20** (1981) 1224.
15. S.K.Chaudhuri, P.S.Roy, A.K.Ghosh, *Indian J.Chem.* **A22** (1983) 996
16. P.G.Harrison, T.J.King, R.C.Phillips, *J.Chem.Soc.Dalton Trans.* (1975) 826.
17. P.G.Harrison, T.J.King,R.C.Phillips, *J.Chem.Soc.Dalton Trans.* (1976) 2317.
18. P.G.Harrison, T.J.King, *J.Chem.Soc.Dalton Trans.* (1974) 2298
19. P.G.Harrison, T.J.King, K.C.Molloy, *J. Organomet. Chem.* **185** (1980) 199.
20. a) T.V.Drovetskaiya, N.S.Yashina, T.V.Leonova, V.S.Petrosyan, J.Lorberth, S.Wocadlo, W.Massa, J.Pebler, *J.Organomet. Chem.* **507** (1996) 201
    b) V.S.Petrosyan, N.S.Yashina, T.V.Sizova, T.V.Leonova, L.A.Aslanov, A.V.Yatsenko, L.Pellerito, *Appl. Organomet.Chem.* **8** (1994) 11
21. T.P.Lockhart, W.F.Manders, *Inorg.Chem.* **25** (1986) 892.
22. H.C.Clark, V.K.Jain, R.C.Mehrotra, B.P.Singh, G.Srivastava, T.Birchall, *J.Organomet.Chem.* **279** (1985) 385.

**Received: July 6, 1998 - Accepted: July 14, 1998**

